# Textile Strain Sensor Enhancement by Coating Metal Yarns with Carbon-Filled Silicone

**DOI:** 10.3390/polym14132525

**Published:** 2022-06-21

**Authors:** Rike Brendgen, Ramona Nolden, Jasmin Simon, Theresa Junge, Kerstin Zöll, Anne Schwarz-Pfeiffer

**Affiliations:** 1 Research Institute for Textile and Clothing (FTB), Niederrhein Universisty of Applied Sciences, Webschulstr. 31, 41065 Mönchengladbach, Germany; ramona.nolden@outlook.de (R.N.); jasminsimon97@yahoo.de (J.S.); theresa.junge@stud.hn.de (T.J.); kerstin.zoell@hs-niederrhein.de (K.Z.); anne.schwarz-pfeiffer@hs-niederrhein.de (A.S.-P.); 2 Faculty of Textile and Clothing Technology, Niederrhein University of Applied Sciences, Webschulstr. 31, 41065 Mönchengladbach, Germany

**Keywords:** strain sensing, yarn coating, electrical conductive polymer coating, gauge factor, composite yarn, hybrid yarn, interlooped yarns

## Abstract

Flexible and stretchable strain sensors are an important development for measuring various movements and forces and are increasingly used in a wide range of smart textiles. For example, strain sensors can be used to measure the movements of arms, legs or individual joints. Thereby, most strain sensors are capable of detecting large movements with a high sensitivity. Very few are able to measure small movements, i.e., strains of less than 5%, with a high sensitivity, which is necessary to carry out important health measurements, such as breathing, bending, heartbeat, and vibrations. This research deals with the development of strain sensors capable of detecting strain of 1% with a high sensitivity. For this purpose, a total of six commercially available metallic yarns were coated with a carbon-containing silicone coating. The process is based on a vertical dip-coating technology with a self-printed 3D coating bath. Afterwards, the finished yarns were interlooped and stretched by 1% while electrical resistance measurements were carried out. It was shown that, although the coating reduced the overall conductivity of the yarns, it also improved their sensitivity to stress. Conclusively, highly sensitive strain sensors, designed specially for small loads, were produced by a simple coating set-up and interlooping structure of the sensory yarns, which could easily be embedded in greater textile structures for wearable electronics.

## 1. Introduction

The growing demand for soft electronics promotes the development of smart textiles. Of particular interest are stretchable, skin-mountable and wearable strain sensors that find application in various sectors, such as personalized health-monitoring [1,2,3,4], human motion detection [5,6,7,8], and human-machine interfaces or soft robotics [9,10,11,12]. Thereby, smart textiles offer the advantages of intuitive interaction with the human body and long-term monitoring capabilities [13]. Strain sensors convert physical deformation into an electrical signal [14,15]. Most textile strain sensors work on a capacitive or resistive principle [15]. Capacitive sensors are based on a sandwich structure, consisting of a dielectric layer in between two electrodes. Under load, the distance between electrodes alters, which is reflected in a change of capacitance [15,16]. Capacitive textile strain sensors were developed for applications including human motion tracking [17,18] and respiration monitoring [19]. Resistive sensors detect strain as a change in the electrical resistance of an electrical conductive material; they are advantageous over others in the diverse selection of possible materials due to their simple structures and suitability to a comprehensive range of applications [15,16]. Resistive, as well as capacitive, sensors require electrical conductive materials. As most conventional textile materials are isolators, they must extrinsically be modified by adding or incorporating conductive materials. Extrinsic conductivity can be achieved by coating or integrating conductive fillers, such as carbon black [20,21,22,23], carbon nanotubes [24], or metallic additives [25,26,27], into the polymer matrix. Intrinsic conductivity, on the other hand, describes materials that are self-conducting and do not need additional processing. These include conductive polymers, such as poly(3,4-ethylenedioxythiophene), polystyrene sulfonate (PEDOT:PSS), or polyaniline (PANI), but also metallic fibers and yarns. Various combinations of polymers, fillers, and metals are also conceivable and should be selected application-specifically [28]. Many strain sensors have been reported as having a large strain range and high sensitivity; however, sensors having a high sensitivity at small strain ranges (< 5%) are far less documented [16]. Therefore, textile strain sensors are often used for motion detection with large strain ranges [6,7,16,23,29,30,31,32,33,34,35,36,37]. Zhao et al., on the other hand, developed a strain sensor with small strain ranges made of a carbon nanotube yarn. The sensory yarn had a sensing range of 1% but low sensitivity, with a gauge factor of approximately 0.5 [38]. Liu et. al. developed a strain sensor capable of detecting feeble human motions at a strain of 3% [39]. Wu et al. developed a strain sensor by coating a polyurethane yarn with a conductive polymer composite layer, consisting of carbon black and natural rubber, that had a gauge factor of 39 and a detection limit of 0.1% strain [40]. Additionally, Wajahat et al. reported on a flexible strain sensor made of a carbon nanotube polyvinylpyrrolidone composite, which showed a GF of 13.07 at 0.8% strain [41]. Despite these findings, flexible/textile strain sensors with a high sensitivity to little changes in loading are still exceptions. Therefore, there is a need for easy-production, highly sensitive textile strain sensors for little loading, as small strains may provide important information about people’s health conditions, e.g., heartbeat, vibrations, torsions, or bending [16]. This lack of easy-production, highly-sensitive textile based sensors at small strains could be filled by the herein presented hybrid yarns, produced from metallic yarns and coated with a carbon-black-filled silicone arranged in an interlooped structure. This combination of conductive components unites the benefits of both materials, which are the high electrical conductivity of the metallic yarns and the high compressibility and flexibility of the carbon-black-filled silicone. The functionality of resistive strain sensors is based on the following equation [15]:

(1) $$R=\rho \;\times \;\frac{L}{A}$$

**Figure 1:**
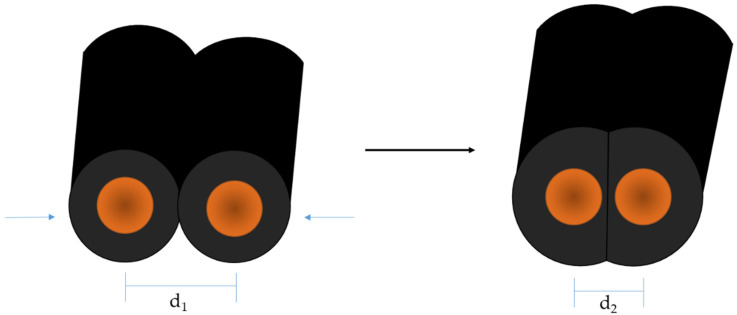
Schematic cross-section of carbon-filled, silicone-coated metal yarn and their deformation upon strain that causes the change in electrical resistance.

Thereby, R is the electrical resistance resulting from the specific electrical resistance of the material (*ρ*) multiplied by the divided length of the distance between the measuring electrodes (*L*) and the cross-sectional area of the sample (*A*). In order to cause a variation in electrical resistance, it is necessary that the load on the sensor changes either the geometry (*L/A*) and/or the specific electrical resistance of the material (*ρ*). The functional mechanism of most resistive strain sensors is based on a complex interaction of several factors, which depend on the structure of the textile, the manufacturing method, and the components used [15,42]. The developed carbon-filled, silicone-coated strain sensors are also subjected to that law, and their electrical resistance changes as the distance between the interlooped yarns decreases upon loading (Figure 1). The compressibility of the carbon-filled silicone reinforces the change in geometry and ensures the high sensitivity of the developed sensory yarns upon loading. The electrical performances, upon strain, of the developed hybrid yarns are compared to each other and to that of the uncoated base material. The sensor characteristics are also analyzed.

## 2. Materials and Methods

Electrical conductive yarns were coated with a carbon-black-filled silicone (Elastosil LR3162, Wacker Chemie AG, Munich, Germany) in a simple, vertical, dip-coating set-up. Subsequently, the samples were characterized optically (VHX-600, Keyence, Mechelen, Belgien) and their electrical performance (Resistomat 2316/clamping device 2381, Burster Präzisionsmesstechnik GmbH & Co. Kg, Gernsbach, Germany) was investigated with regard to the change in electrical resistance as a function of strain. The factor of resistance change as well as the Gauge-Factor were calculated from the electrical measurements.

### 2.1. Materials

In total, six commercially available metallic yarns were coated with carbon-black-filled silicone (Elastosil LR3162, Wacker Chemie AG, Munich, Germany). In addition, the silicone oil Belsil DM 1 Plus (Wacker Chemie AG, Munich, Germany) was used to adjust the viscosity of the coating mixture.

Following substrates were chosen (Table 1):

### 2.2. Methods

The electrical conductive yarns (Table 1) were coated in a simple dip-coating set-up that allows coating of diverse, yarn-like substrates by a 3D-printed nozzle with attachable dosing needles. Afterwards, the produced hybrid yarns were characterized for their layer thickness and homogeneity of the coating, as well as for their electrical properties.

#### 2.2.1. Coating of the Metallic Yarns

The coating dispersion was prepared by mixing the carbon-filled silicone and the silicone oil in a ratio of 1:1. Subsequently, both prepared components were mixed together in equal amounts.

**Figure 2:**
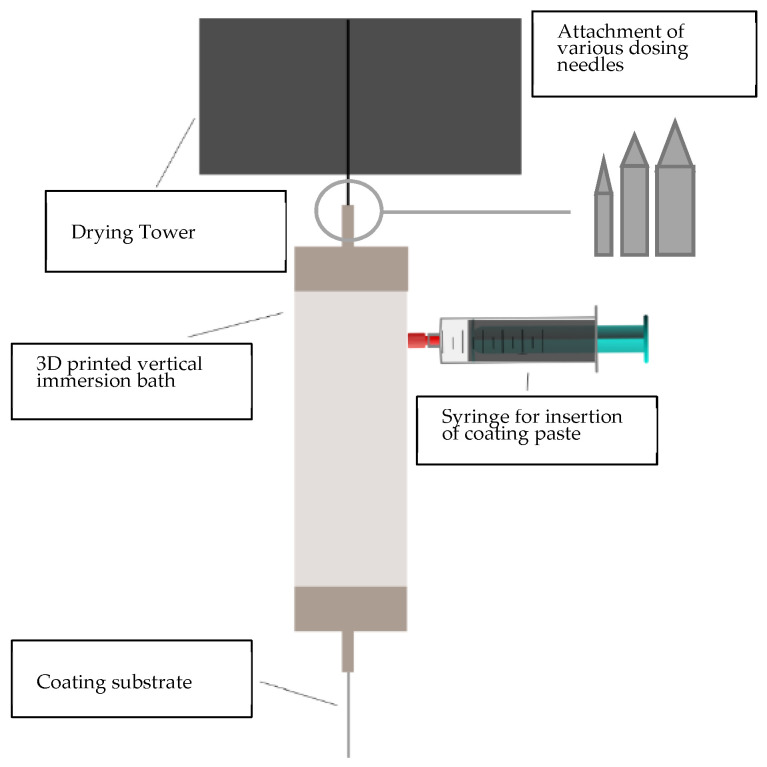
Schematic drawing of coating set-up.

The coating process is based on vertical dip-coating technology. A similar coating set-up has been previously published [43]. Herein, a 3D-printed immersion bath was deployed in the yarn drying tower FMP-Ditro 3D from FMP Technology GmbH (Erlangen, Germany). Dosing needles can be attached to the immersion bath, so that different outlet openings, depending on the substrate, can be realized. In this coating set-up, a dosing needle with a diameter of 1.12 mm (supplier VIEWEG GmbH, Kranzberg, Germany) was chosen for all substrates. Figure 2 shows the schematic drawing of the coating set-up. The substrate is unwound vertically and passes through the coating bath, while coating paste is injected. When exiting the coating bath, it directly enters the drying tower; at its exit, the substrate gets redirected and finally wound up by a winding unit with adjustable winding speed. The process parameters were set at a 165 °C drying temperature and a process speed of 0.2 m/min. The drying tower has a height of 2.0 m, so that the substrate remained for 10 min in the drying section.

#### 2.2.2. Optical Microscopy

Optical microscopic examinations were performed using a VHX-600 from Keyence (Mechelen, Belgien). The lens VH-Z20R was used, with a magnification area of 20–200 times for longitudinal views. The lens VH-Z250R, with a magnification area of 250–2500 times was used for cross-sectional views. The cross-sectional micrographs were also used to identify the layer thickness of the coating.

#### 2.2.3. Electrical Resistance Measurements

**Figure 3:**
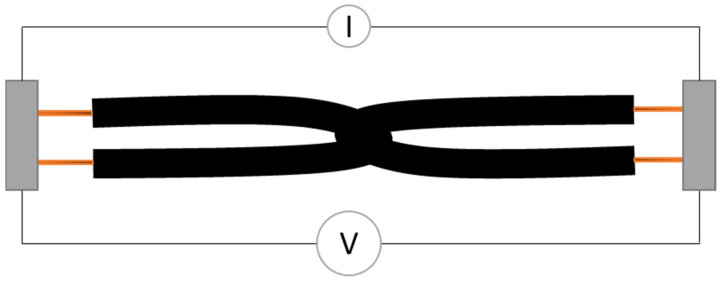
Schematic drawing of four-point probe method carried out at the interlooped hybrid yarns.

The electrical properties of the samples were examined by measuring the surface electrical resistance applying a four-point-probe method (Figure 3). Compared to the traditional, two-point sensing method, the four-point-probe method uses separate pairs of current-carrying and voltage-sensing electrodes to improve accuracy, as contact resistance can be compensated for [44]. The device used was a Resistomat 2316 with the clamping device type 2381 (Burster Präzisionsmesstechnik GmbH & Co. Kg, Gernsbach, Germany). Calculations of the theoretical electrical resistance are omitted for the following reasons: textiles in general, which comprise yarns as well as fabrics, are inhomogeneous and anisotropic products. Therefore, the electrical properties vary depending on the raw material, its structure, and geometrical dimensions. The anisotropy of flat textile materials has been described in many articles [45,46,47], but the same observations also hold true for yarn-based, electrical conductive textiles. The used yarns are made of single silver, copper-coated filaments, or steel filaments that are twisted with each other. Thereby, not only the coating of the individual filaments is irregular, but also the total cross-section of the yarns caused by the twisting. Consequently, the carbon-filled silicone coating is also applied inhomogeneously and not in a perfectly round manner. Theoretical calculations of the electrical resistance of those hybrid yarns, therefore, become difficult and the surface electrical resistance was obtained simply through measurements.

**Figure 4:**
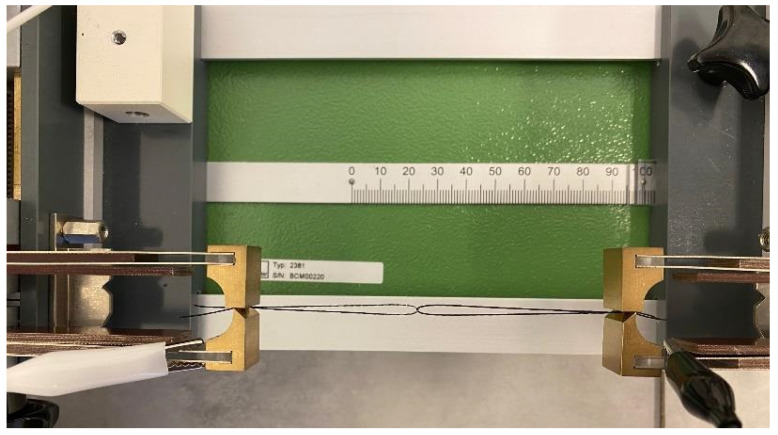
Image of the real measurement set-up of the interlooped sensory yarns.

In order to measure the electrical resistance of the metal yarns under compressive loads, the test set-up of the measurement followed the standard “DIN 53843-1-Loop tensile test”. The loop tensile test provides that two sections of a specimen are intertwined and clamped so that the total length amounts to 100 mm. One clamp is then moved by one millimeter so that the specimen is stretched by 1% and thus loaded. Subsequently, the clamp is moved back to a length of 100 mm. Stretching and releasing of the sample corresponds to one cycle. The samples were prepared in such a way that the coating was removed from the ends, where the samples were clamped into the set-up. The electrical contacts were therefore on the metallic yarns, rather than on the coating. This ensures that the change of electrical resistance due to compression of the substrates caused by load is measured and not the changes of resistance on the surface of the coating. From those measurements, the factor of resistance change as well as the Gauge-factor were calculated. The real measurement set-up, consisting of the sliding clamping device and the interlooped structure of the sensory yarns, can be seen in Figure 4.

## 3. Results

The layer thickness as well as the homogeneity of the coating were analyzed optically and revealed that the coating process was capable of producing a homogenous layer over a long distance but not continuously. The electrical measurements show that the coating improved the sensitivity of the yarns towards loading and worked best on electrical conductive sewing threads (Shieldex, Silvertech, Silvertech+).

### 3.1. Layer Thickness

**Figure 5:**
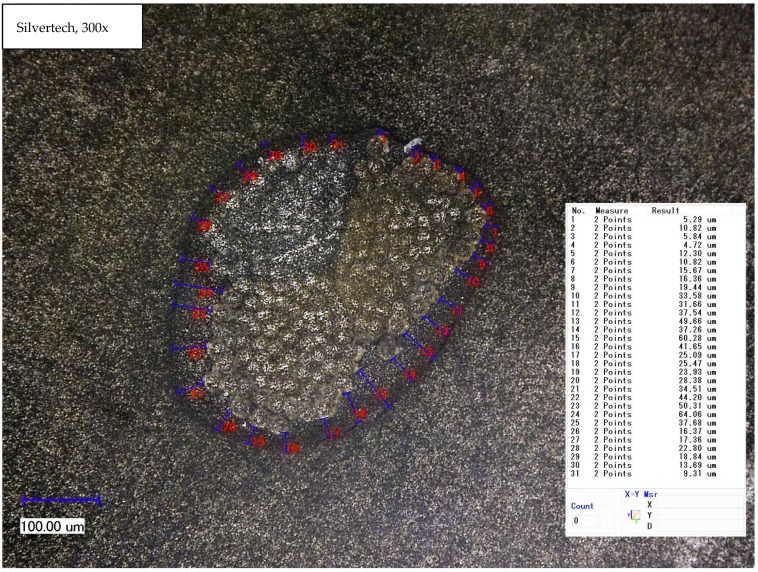
Light microscopic measurement of the layer thickness of the coating exemplarily here at the Silvertech substrate.

From the cross-sectional samples in Section 3.2.2. one can approximately determine the layer thickness of the coating, which is shown here exemplarily in Figure 5. Measurement points from the outer coating to the outer edge of the yarn were set and the distance was measured by the software of the microscope. Calculating the mean from all measurement points, the layer thickness of the coated Silvertech yarn amounts to 26.60 µm.

Table 2 sums up the mean values of the measurements carried out on each substrate. Thereby, Bekinox, Highflex 3981, and Highflex 7077 had a significantly higher coating thickness than the other substrates, Shieldex, Silvertech, and Silvertech+. Highflex 7077, with 54.44 µm, had the highest and Silvertech+, with 21.85 µm, had the lowest coating thickness.

### 3.2. Optical Evaluation of the Samples

The coating was characterized optically and, thereby, longitudinal as well as cross-sectional views were taken. They provided insight about the different structures of used substrates and the coating morphology.

#### 3.2.1. Longitudinal View

**Figure 6:**
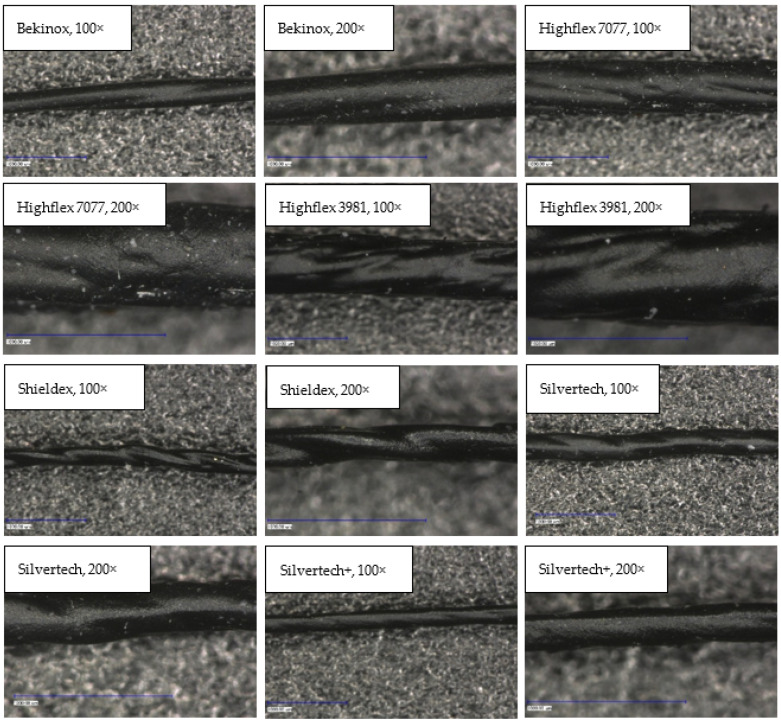
Light microscopic images of coated yarns.

First examinations of the samples were carried out by light microscopy and provide information about the coating morphology. Figure 6 shows the longitudinal view on the samples at 100-times and 200-times magnification. It becomes clear that a uniform coating depended significantly on the metallic substrate’s material and structure. The coating covered the yarns while the textile character, especially the twisting, was still recognizable. As it is shown in the micrographs in Table 1, the Shieldex yarn was twisted most. Even after coating, this inherent structure was clearly identifiable. Also, the clear size difference between the Highflex 7077 and the Highflex 3981, compared to the other yarns, became visible.

#### 3.2.2. Cross-sectional View

**Figure 7:**
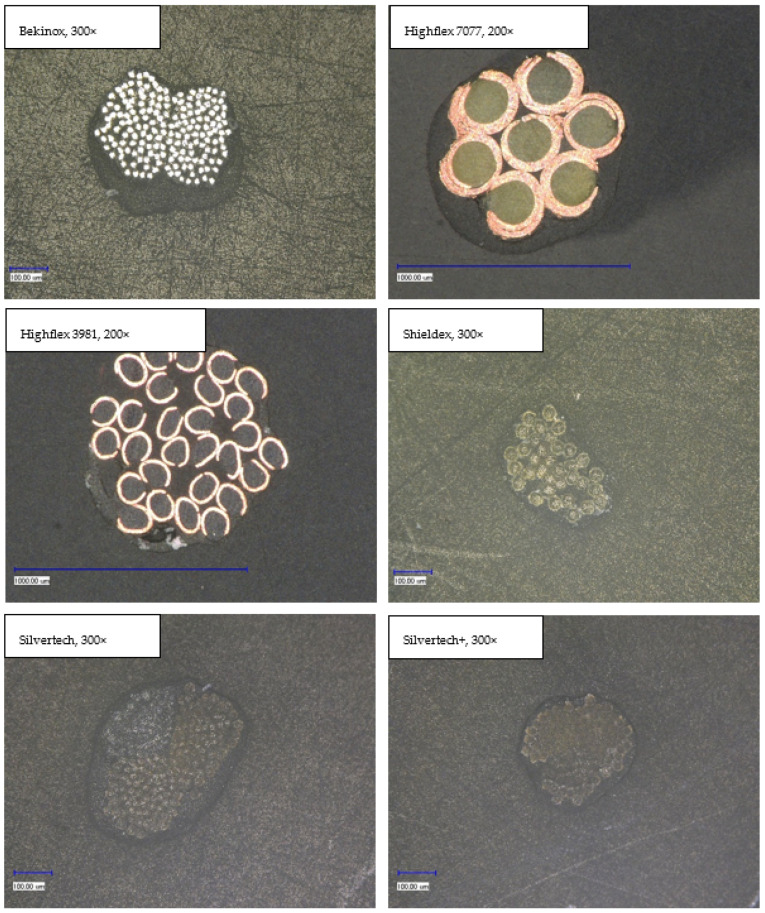
Cross-sectional view of coated samples showing the sheathing of substrates with the coating film.

In order to be able to make further statements about the coating layer quality, cross-section samples were made by resining. Figure 7 shows the cross-sectional view of each substrate; while most images were taken at 300-times magnification, the Highflex 7077 and Highflex 3981 were taken at 200-times magnification as they are bigger in size than the other substrates. All substrates were clearly surrounded by the black coating, which did not penetrate into the substrate structure. This can be explained by the high viscosity of the coating paste, which caused the capillaries between the single fibre strands not to be reached by the paste. The coating was not always uniformly applied around the yarn. That was especially visible at the Bekinox and Highflex 7077 yarn: one side contained far more coating material. Additionally, the different structures of the yarn substrates became visible; while the Bekinox, Shieldex and Silvertech yarns were made of multiple fiber strands, the Highflex 7077 was simply made of seven wrapped filaments. The finer yarns seem to be more compressible by the applied coating as they took on an oval shape, while the Highflex 7077 kept its round form. Furthermore, the bigger diameter of the single fiber strands of the Highflex 7077 yarn created larger indentations on the outer side of the yarn that are filled with coating material. This is reason for the higher mean carbon filled silicone coating take-up depicted in Table 2.

### 3.3. Resistance Measurements

Resistance measurement were carried out to examine the electrical characteristics of the samples and their sensitivity to load. For that, each substrate was tested on the basis of five samples that underwent four cycles of loading and unloading. For comparative purposes, the same measurement was carried out using the uncoated substrates.

**Figure 8:**
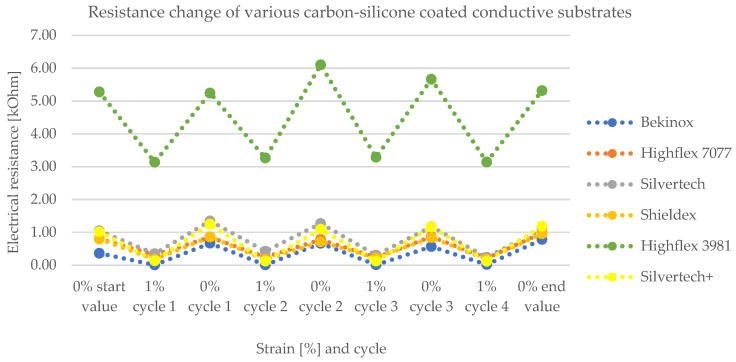
Change of electrical resistance of various carbon-silicone coated substrates upon loading and unloading, showing the working principle of the sensory yarn as electrical resistance decreases upon 1% loading.

**Figure 9:**
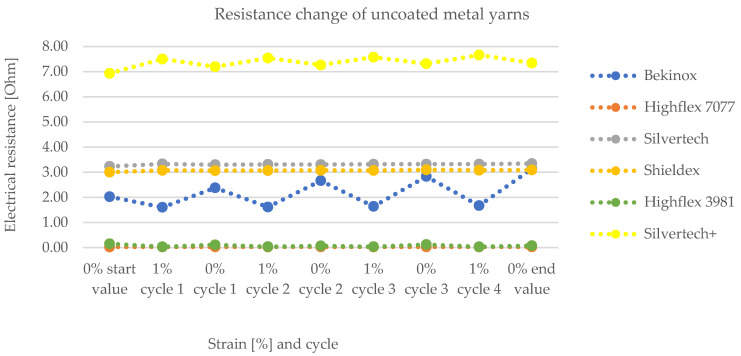
Change of electrical resistance of uncoated substrates upon loading and unloading showing too few changes during load cycles.

**Figure 10:**
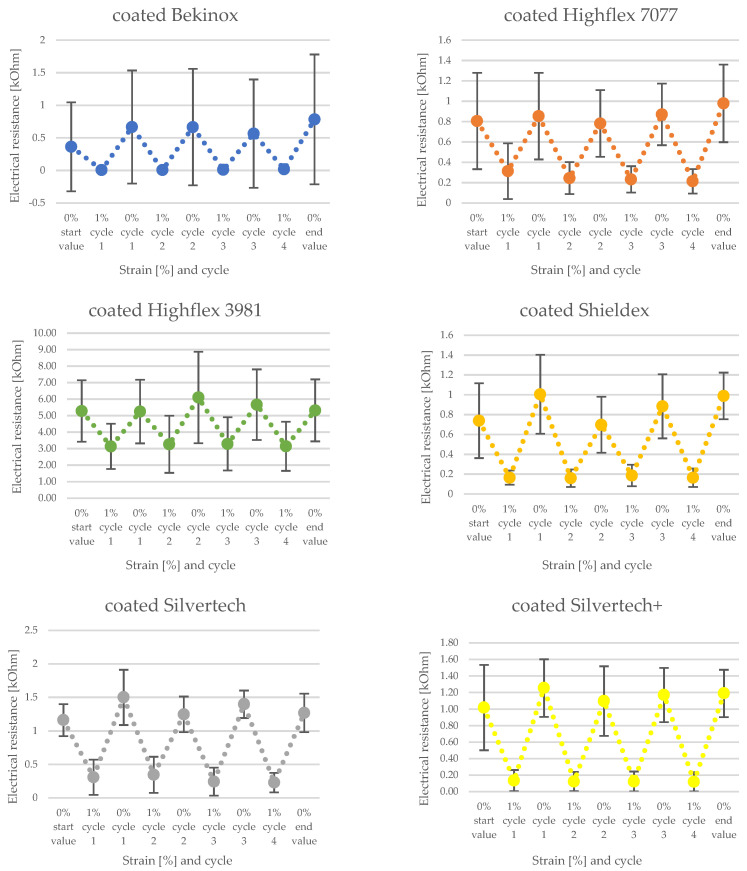
Single display of change of electrical resistance of coated substrates with standard deviation.

**Figure 11:**
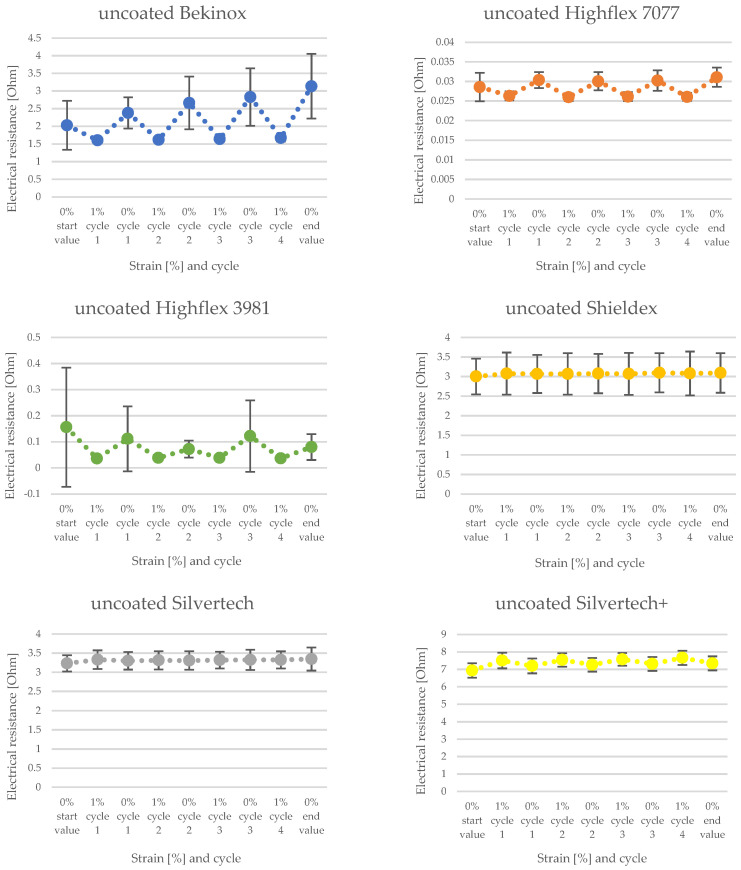
Single display of change of electrical resistance of uncoated substrates with standard deviation.

Figure 8 and Figure 9 show the change of electrical resistance upon four cycles of loading and unloading of all substrates, whereof Figure 8 includes the coated and Figure 9 the uncoated substrates tested with the same measurement set-up. In Figure 8, the carbon-silicone-coated Bekinox yarn shows the lowest and the coated Highflex 3981 yarn shows the highest electrical resistance values. The size range of all samples was comparable, though the Highflex 3981 had approximately 5 times higher resistance values than the other substrates. The Bekinox yarn initially showed an electrical resistance value of 0.3638 kOhm. When it was loaded about 1%, electrical resistance decreased to 0.0078 kOhm. For the Highflex 3981 yarn, values are as follows: the initial resistance lay at 5.2792 kOhm but, upon loading, it dropped down to 3.1372 kOhm. When comparing Figure 8 and Figure 9, it must be noted that for the uncoated samples the resistance values are given in Ohm. Thus, the coating increased the resistance approximately one-thousand-fold. However, the amplitudes of the change of electrical resistance upon loading of the uncoated samples were lower. Here, the Highflex 7077 had the lowest electrical resistance values, starting at 0.0286 Ohm and decreasing to 0.0263 Ohm upon loading. The Silvertech+ yarn had the highest value, given at 6.9318 Ohm, and increased upon loading, unlike all previous samples, to 7.0522 Ohm. The single displays of the measurements, including the standard deviation, are given in Figure 10 for the coated and in Figure 11 for the uncoated samples. For coated and uncoated substrates, it is observable that the values during loading were mostly more consistent over all cycles than the values during unloading. This becomes apparent when looking at the standard deviations in Figure 10 and Figure 11. For the coated Shieldex substrate exemplary, the standard deviation of unloading lies between 0.25 and 0.49, whereas the standard deviation of loading lies between 0.06 and 0.08. The effect of the coating regarding the sensitivity of the sensor becomes especially apparent when comparing the uncoated Shieldex, Silvertech, and Silvertech+ yarns to their coated equivalents. The uncoated substrates show nearly no change in electrical resistance when loaded or unloaded while the coated substrates triple to tenfold their values during the load cycles. The uncoated Bekinox, Highflex 7077, and Highflex 3981 already show sensor characteristics in their uncoated form, as the electrical resistance dropped during loading. Nevertheless, the sensitivity of the yarns towards loading can still be enhanced by the coating. The only exception is the Highflex 3981, which showed better sensor properties in the uncoated state.

The sensitivity of the substrates can be clarified by the factor of the resistance change. It is calculated as follows in Formula (2):

(2) $$F=\frac{R_{0}}{R}$$

**Figure 12:**
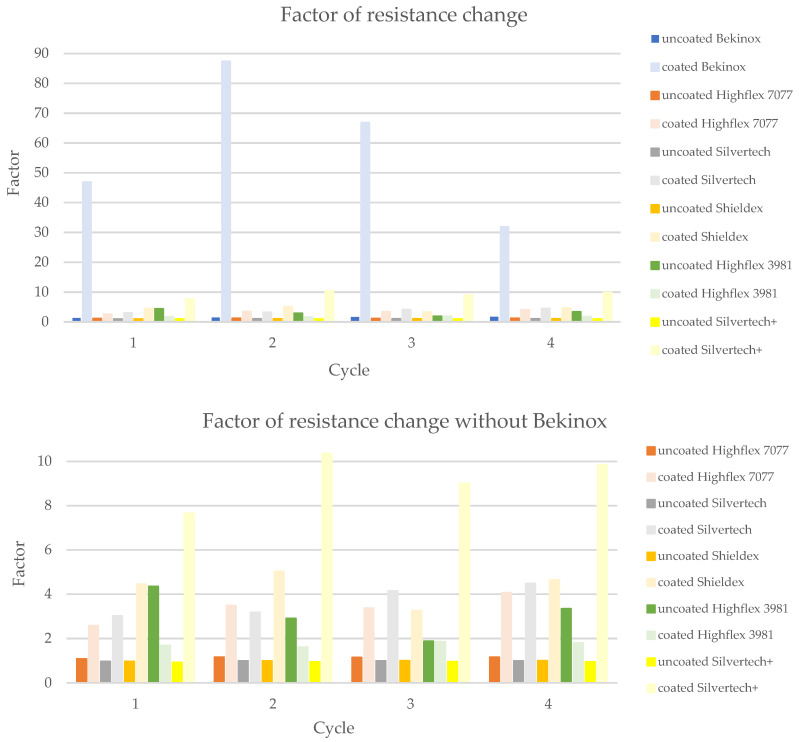
Factor of resistance change of the uncoated substrate as comparison to the coated substrates to illustrate the influence of the coating on the sensitivity of the yarns.

The corresponding factors for the tested uncoated and coated samples are given in Figure 12. Herein, the uncoated substrates are depicted in full color tones, whereas the coated equivalents are displayed in pastel tones. Due to the remarkably low resistance values during loading of the coated Bekinox samples (see Figure 10, coated Bekinox), the factor of resistance change was rather high; in the second cycle, for example, the factor was 87.41 (Figure 12, light blue). For comparison, the factors of the other samples lay between 1.5 and 10.0 (Figure 12). Since the Bekinox specimen dropped out, the other samples are depicted in Figure 12 (bottom) without Bekinox. Here, it becomes apparent that the coating increased the factor of resistance change and, thereby, the sensitivity of the sensory yarns, except for the Highflex 3981. All pastel colours, except of Highflex 3981 in pastel green, are higher than their full-colour equivalents, and the coated Silvertech+ in pastel yellow shows a particularly large increase. In the second cycle, for example, the factor of resistance change increased from 0.9542 (yellow) to 10.3444 (pastel yellow), which can be attributed to the coating.

The significance of the factor of resistance change of the coated Bekinox sample must be questioned when looking at its standard deviation in Figure 9. It is obvious that the deviation was higher than the actual values, at least during unloading. While the actual values lay between 0.36 and 0.78 kOhm, the standard deviation for those values was given by 0.62 and 1.00. The standard deviation of the coated Highflex 7077 was rather high too, though the deviation did not exceed the actual values. However, the deviations downwards and upwards between unloading and loading overlapped or, respectively, were rather close, so that the reliability of this sensory yarn is not given. The same observation held true for the coated Highflex 3981 (Figure 10); consequently, the working principle must be viewed critically. The standard deviation for the uncoated equivalents to Bekinox, Highflex 3981, and Highflex 7077 was rather high too (Figure 11); therefore, the cause of this high deviation must already be sought in the raw material and the suitability of these substrates for this measurement setup should be generally questioned.

**Figure 13:**
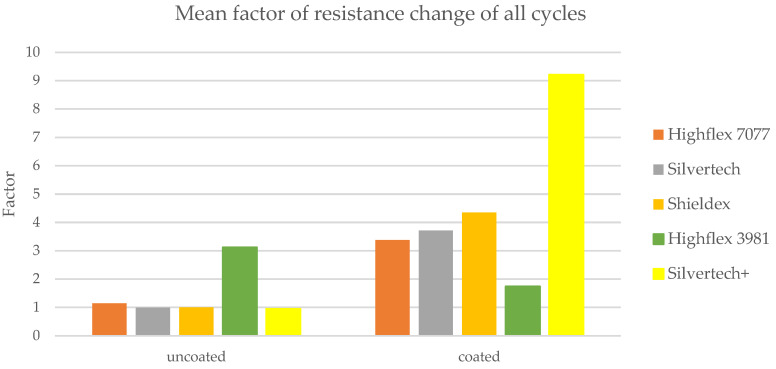
Mean factor of resistance change of all cycles demonstrating the enhancement of sensory properties towards loading by coating.

Figure 13 shows the calculated mean values of the factors measured during the different cycles, whereby the Bekinox sample is not depicted due to the described issue. In this simplified diagram, the increase of the factor becomes particularly apparent. The highest rise was recorded for the Silvertech+ samples, but Silvertech, Shieldex, and Highflex 7077 also show significant increases. The coating on the Highflex 3981, on the other hand, caused exactly the opposite: a decrease of the factor of resistance change was observed for the coated samples compared to their uncoated equivalents.

Next to the factor of resistance change, one can calculate the Gauge-factor, which is the magnitude of the resistance change over applied strain and can be calculated by Formula (3) [15]:

(3) $$GF=\frac{\Delta R/R_{0}}{\varepsilon }$$

*R_0_* is thereby the resistance of the sensor at the initial unstretched state, Δ*R* is the difference between the resistance between the stretched state and *R_0_* and *ε* are the applied strain ratio. A high *GF* value means a highly sensitive sensor [15]. A negative *GF* describes that resistance decreases when tensile stress is applied. Table 3 sums up the calculated mean Gauge-factors of all test cycles of the individual substrates, distinguishing between the uncoated and coated specimens. The findings described above can thus be substantiated once again. The coated Bekinox samples had the highest GF, of −98.2304. The GF of the Highflex 3981 was reduced upon coating the substrate from −65.5768 to −41.9042. The highest increase of GF upon coating can be found for the Silvertech+ sample, whereby the uncoated substrate had a GF of 4.9875 and the coated equivalent of −89.0990. The remaining substrates, Highflex 7077, Silvertech and Shieldex, had GF values of around 70 when coated.

## 4. Discussion

The results of microscopic measurement of the coating thickness are surprising, as the thicker metallic yarns, especially Highflex 3981 and Highflex 7077, had a higher coating thickness with the same dosing needle opening than the smaller substrates. Logically, one would expect the thinner yarns to have a thicker coating thickness as they take up less space that can be filled with coating dispersion in the dosing needle opening. The reason for the greater coating compound take-up must therefore lie in the structure of the metallic substrates. Shieldex, Silvertech, and Silvertech+ are conductive sewing threads that are flexible, bendable, and compressible. Bekinox, Highflex 3981, and Highflex 7077 are stiff materials and (especially the Highflex yarns) are significantly larger but still composed of fewer, yet thick, filaments. These thick filaments formed distinct depressions into which the coating mass was deposited and which caused the high averaged coating thickness. Contrarily, the Shieldex, Silvertech, and Silvertech+ yarns are compressible; the coating could be applied more evenly around the entire yarn.

The microscopic examination of the coated yarns revealed weaknesses in the coating process, though generally it is possible to apply the high-viscosity carbon silicone coating with this simple coating set-up. To guarantee a uniform coating, sufficient dosage should always be ensured. Over long distances, the coating was evenly applied, while the textile character of the yarns was still visible. The cross-sectional view clearly illustrates the different yarn compositions and sizes of yarns and individual filaments.

The electrical resistance measurements reveal that the uncoated metallic sewing threads (Shieldex, Silvertech, Silvertech+) demonstrate no sensitivity towards loading. Upon 1% stretching of the interlooped yarns, little to no change in electrical resistance was detected. The stiffer and coarser yarns (Bekinox, Highflex 7077, Highflex 3981), however, showed sensitivity towards loading as their electrical resistance values alternately decreased and increased during the loading cycles. The reason for the different electrical properties must lie in the structure of the used yarns. The flexible metallic sewing threads yielded to the load and, thus, showed no change in their electrical properties, whereas the stiffer materials were deformed during loading, which resulted in decreased electrical resistance values. In order to enhance the sensitivity of the metallic yarns towards loading, a carbon-silicone coating was applied. As it was a high-resistance coating, the overall electrical resistance of all coated substrates increased one-thousand-fold, so that the measuring unit is depicted in Ohm for the uncoated samples and in kOhm for the coated samples. The contacting of the interlooped coated yarns ran across the coating, which is reason for the higher electrical resistance values. While the overall resistance of the samples increased, the sensitivity of the samples was enhanced. The sensitivity of a sensor can be expressed as a factor that describes the change in electrical resistance during load cycles. Thereby, a factor close to 1.0 stands for low sensitivity, whereas a higher factor stands for better sensitivity. Coating the conductive sewing threads (Shieldex, Silvertech, Silvertech+) increased the mean factor of resistance change, from 0.9782 for the uncoated samples to 5.7551. Thereby, best results could be achieved with the coated Silvertech+ samples, whose factor reached as high as 10.3444. These findings are also proven by the calculated Gauge-factor, which was increased from 4.9875 for the uncoated Silvertech+ sample to −89.0990 of the coated equivalent. Note that the negative prefix indicates only that resistance decreases when tensile stress is applied. While the coating clearly improved the sensor properties of the flexible conductive sewing threads (Shieldex, Silvertech, Silvertech+), the same effect could not be observed for the coarser and stiffer conductive yarns (Bekinox, Highflex 7077, Highflex 3981). As for the Highflex 3981, the factor of resistance change as well as the Gauge-factor was actually reduced after coating the samples. For the Bekinox and Highflex 7077 samples, the factors could be increased by coating, but the measurements showed extreme variations, especially for the Bekinox yarn. Those variations could be attributed to the irregular coating, which was noticed during microscopic examination. Additionally, the coating enhanced the stiff properties of the samples, so that stretching became more difficult, which also was a reason for the deteriorated sensor properties. In conclusion, the coating on the stiffer materials (Bekinox, Highflex 7077, Highflex 3981) did not improve the sensory characteristics as imagined, but had a huge effect in terms of sensitivity on the flexible conductive sewing threads (Shieldex, Silvertech, Silvertech+). Table 4 sums up the findings of the examinations for a faster comparison of the coated and uncoated substrates.

## 5. Conclusions

Many strain sensors have been reported having a large strain range and high sensitivity, however sensors having a high sensitivity at small strain ranges (< 5%) are far less reported. Those small strain ranges are of interest for small body movements such as heart beat or respiration. Therefore, we successfully developed a textile-based sensor that was capable of detecting small strains (1%) with a high sensitivity. Various commercially available, electrically conductive yarns were dip-coated with a carbon-containing silicone in a self-printed 3D nozzle and subsequently dried. The coated yarns were then interlooped and stretched by 1 %, so that the contact area between the interlaced yarns changed. The coating reduced the overall conductivity of the samples as it was a high resistance coating, though it improved the sensitivity of the yarns towards loading. The coating worked best on flexible conductive yarns, such as the sewing threads Shieldex, Silvertech, and Silvertech+. In this way, the mean factor of resistance change of those samples could be increased from 0.9782 to 5.7551. On the other hand, stiffer and coarser materials such as Bekinox, Highflex 3981, and Highflex 7077 showed significantly more irregularities, deteriorated haptic properties, and worsened sensor characteristics after coating. Nevertheless, highly sensitive strain sensors especially for small loads were produced by this simple coating set-up and interlooping structure of the sensory yarns, which could easily be embedded in greater textile structures for wearable electronics.

## Figures and Tables

**Table 1:** Substrate metallic yarns for coating with conductive silicone.

Name	Manufacturer	Composition	Resistance	Fineness (Dtex)	Light-Microscopy Image
Bekinox	NV Bekaert SA (Zwevegem Belgium)	Stainless steel	29 Ohm/m	2500	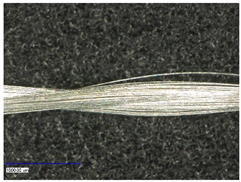
Highflex 7077	Karl Grimm GmbH & Co. KG (Roth, Germany)	Silver-plated copper, Carrier material: Kevlar	0.41 Ohm/m	N/A	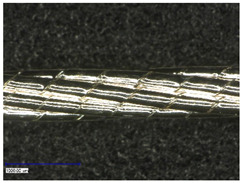
Highflex 3981	Karl Grimm GmbH & Co. KG (Roth, Germany)	Copper, carrier material: polyethylene terephthalate	0.55 Ohm/m	N/A	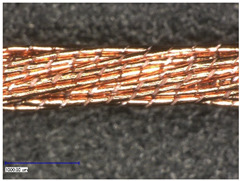
Shieldex	Statex Produktions- und Vertriebs GmbH (Bremen, Germany)	Silver-plated polyamide multifilament yarn	< 300 Ohm/m	295	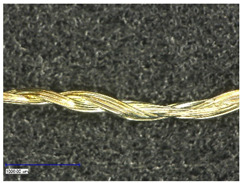
Silvertech	Amann & Söhne GmbH & Co. KG (Bönnigheim, Germany)	Silver coated polyamide/polyester hybrid twine	<150 Ohm/m	210 *3	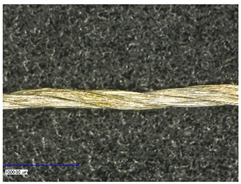
Silvertech+	Amann & Söhne GmbH & Co. KG (Bönnigheim, Germany)	Silver coated polyamide multifilament	< 200 Ohm/m	110 *3	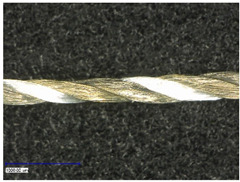

**Table 2:** Coating thickness calculated by averaging light microscopic cross-section measurements of the coating.

Substrate	Mean Carbon Silicone Coating Thickness
Bekinox	46.18 µm
Highflex 7077	54.44 µm
Highflex 3981	41.16 µm
Shieldex	27.25 µm
Silvertech	26.60 µm
Silvertech+	21.85 µm

**Table 3:** Calculated mean Gauge-factor of each substrate, uncoated and coated.

Substrate	Uncoated	Coated
Bekinox	−37.3241	−98.2304
Highflex 7077	−13.0815	−69.6716
Highflex 3981	−65.5768	−41.9042
Shieldex	0.2092	−77.4867
Silvertech	0.5909	−72.2907
Silvertech+	4.9875	−89.0990

**Table 4:** Summary of results.

	Bekinox		Highflex 7077		Highflex 3981		Shieldex		Silvertech		Silvertech+	
	**Uncoated**	**Coated**	**Uncoated**	**Coated**	**Uncoated**	**Coated**	**Uncoated**	**Coated**	**Uncoated**	**Coated**	**Uncoated**	**Coated**
**Coating thickness [µm]**	/	46.18	/	54.44	/	41.16	/	27.25	/	26.60	/	21.85
**Factor of resistance change**	1.5121	58.2359	1.1409	3.3727	3.1184	1.7370	0.9959	4.3439	0.9908	3.7081	0.9480	9.2134
**Gauge factor**	−37.3241	−98.2304	−13.0815	−69.6716	-65.5768	−41.9042	0.2092	−77.4867	0.5909	−72.2907	4.9875	−89.0990
